# Do Only Calcium and Vitamin D Matter? Micronutrients in the Diet of Inflammatory Bowel Diseases Patients and the Risk of Osteoporosis

**DOI:** 10.3390/nu13020525

**Published:** 2021-02-05

**Authors:** Alicja Ewa Ratajczak, Anna Maria Rychter, Agnieszka Zawada, Agnieszka Dobrowolska, Iwona Krela-Kaźmierczak

**Affiliations:** Department of Gastroenterology, Dietetics and Internal Diseases, Poznan University of Medical Sciences, 61-701 Poznań, Poland; a.m.rychter@gmail.com (A.M.R.); a.zawada@ump.edu.pl (A.Z.); agdob@ump.edu.pl (A.D.)

**Keywords:** colitis, ulcerative, inflammatory bowel disease, Crohn’s disease, osteoporosis, micronutrients, silicon, fluorine, zinc, selenium, iron, cadmium, bone mineral density

## Abstract

Osteoporosis is one of the most common extraintestinal complications among patients suffering from inflammatory bowel diseases. The role of vitamin D and calcium in the prevention of a decreased bone mineral density is well known, although other nutrients, including micronutrients, are also of extreme importance. Despite the fact that zinc, copper, selenium, iron, cadmium, silicon and fluorine have not been frequently discussed with regard to the prevention of osteoporosis, it is possible that a deficiency or excess of the abovementioned elements may affect bone mineralization. Additionally, the risk of malnutrition, which is common in patients with ulcerative colitis or Crohn’s disease, as well as the composition of gut microbiota, may be associated with micronutrients status.

## 1. Introduction

More and more people suffer from inflammatory bowel disease (IBD). In 2017, IBD affected 6.8 million people worldwide [1]. In Europe, in 2010, the incidence of ulcerative colitis (UC) and Crohn’s disease (CD) amounted to 9.8 and 6.3 per 100,000 inhabitants, respectively [2]. Moreover, due to their condition, IBD patients are particularly at a risk of developing malnutrition and extraintestinal manifestations [3].

According to the World Health Organization, malnutrition is a deficiency, imbalance or an excess intake of energy or/and nutrients, including micronutrients [4]. In fact, it constitutes one of the risk factors in the development of osteoporosis in patients with IBD [5]. Malnutrition in patients suffering from IBD is multifactorial and has been associated with malabsorption, a decreased calories consumption, pharmacological treatment, nutrient loss in the gastrointestinal tract and an increased energy expenditure [6]. Additionally, malnutrition has also been associated with a poorer quality of life [7].

Calcium and vitamin D are the most frequently discussed nutrients in the prevention of low bone mineral density (BMD) [8]. However, other nutrients, including micronutrients, should also be included in the discussion, since they play an essential role in proper bone mineralization [9,10]. A deficiency of micronutrients does not cause direct clinical symptoms; thus, its diagnosis is challenging in standard laboratory tests. Therefore, a decreased level of micronutrients is often overlooked in establishing the causes of the ailments. Furthermore, a deficiency of micronutrients may be associated with intestinal microbiota [11].

## 2. Nutrition in IBD

**Figure 1:**
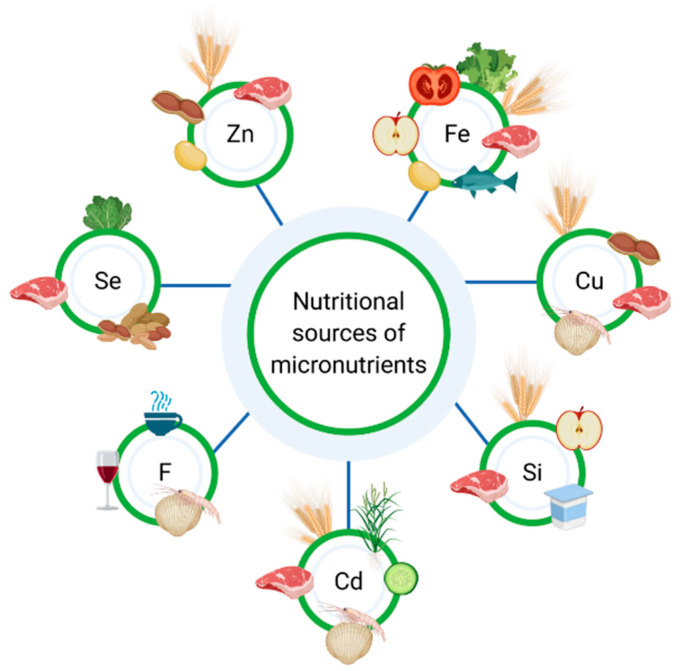
Nutritional sources of micronutrients.

Malnutrition, including the micronutrients deficiency, may affect both UC (ulcerative colitis) and CD (Crohn’s disease) patients, although it is more common among patients with Crohn’s disease. Therefore, IBD patients should be regularly monitored with regard to malnutrition [12]. Calcium and vitamin D are the most commonly discussed micronutrients in terms of osteoporosis prevention [13,14,15,16]; nevertheless, vitamin B12, folic acid, vitamin K, vitamin C, phosphate, magnesium and sodium also play an important role in the prevention of osteoporosis [17]. According to Owczarek et al., the key nutrients for IBD patients comprise iron, calcium, vitamin D, vitamins B, vitamin A and zinc [18], but other components of the diet, such as other micronutrients, should be taken into consideration as well. Figure 1 presents the sources of micronutrients. Moreover, the European Society of Clinical Nutrition and Metabolism (ESPEN) indicate that patients suffering from IBD should be particularly monitored for micronutrient deficiency, since it can affect aspects such as normal growth and bone health [12].

## 3. Osteoporosis in IBD

Musculoskeletal disorders, including osteoporosis, are the most common extraintestinal complications in IBD patients [19]. Osteoporosis constitutes a bone disorder which stems from an imbalance between bone resorption and bone formation [20], and leads to a reduction in bone strength and an increased risk of fractures [21]. It is vital to notice that bone disorders may influence both morbidity and mortality [22]. A gold standard regarding the diagnosis of osteoporosis is dual-energy X-ray absorptiometry (DXA), which is used for the evaluation of the lumbar spine and femoral neck BMD [23].

**Figure 2:**
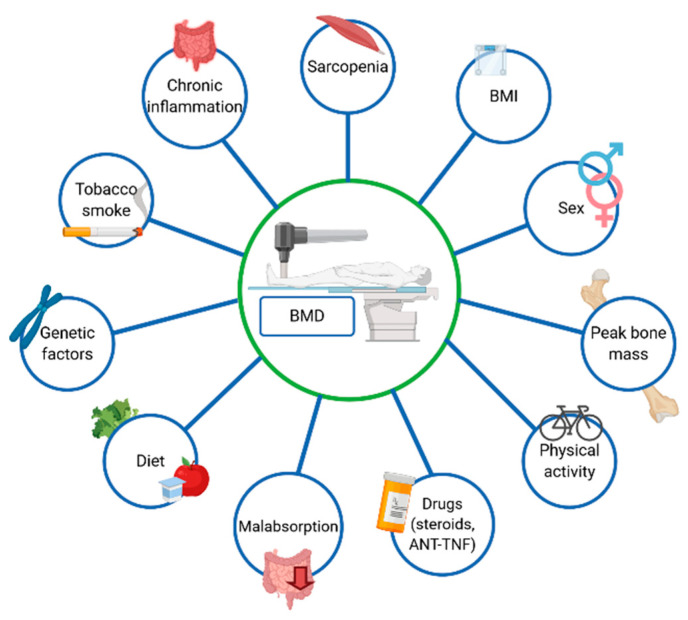
Risk factors of osteoporosis in inflammatory bowel diseases.

Among the newly diagnosed IBD patients, osteoporosis and sarcopenia affects 11% and 46% subjects, respectively [24]. Krela-Kaźmierczak et al. reported that lumbar spine osteoporosis occurred in 11.7% and 3.8% patients with CD and UC, respectively. Additionally, femoral neck osteoporosis was diagnosed in 5.8% of patients with CD and in 2.9% of patients suffering from UC. It is generally accepted that factors affecting BMD include age, gender and peak bone mass. Interestingly, BMD correlates also with the BMI (Body Mass Index) [5], but the main risk factors of osteoporosis in IBD is steroids use. Additionally, chronic inflammation and malabsorption, which lead to a decreased absorption of nutrients and are essential for a proper bone mineralization, also influence BMD [23,25]. Moreover, genetic factors of osteoporosis in IBD have also been discussed [26]. The most vital system responsible for the development of osteoporosis in IBD is probably RANK/RANKL/osteoprotegerin. It is worth bearing in mind that the expression of RANL and osteoprotegerin depends on the single nucleotide polymorphism. However, Krela-Kaźmierczak et al. demonstrated that the molecular background of osteoporosis in CD and UC is different [27] Additionally, the concentration of pro-inflammatory cytokines (e.g., TNF-α) are increased among patients suffering from IBD, which, in turn, might affect BMD [28]. Furthermore, the risk factor of the development of osteoporosis is physical inactivity [29], whereas patients suffering from IBD often avoid exercise due to the gastrointestinal symptoms. In addition, cigarette smoking also affects bones, as cigarette smoke contains more than 7000 chemicals, including cadmium, which may be detrimental to the bone tissue [30]. Fortunately, in the recent years, the prevalence of cigarette smoking among IBD patients has reduced in Western countries [31]. It has been well established that the steroids and biopharmaceuticals used in the IBD therapy also affect bone mineral density [17]. In fact, Sole reported that infliximab increased BMD of the femoral neck in patients suffering from rheumatoid arthritis [32]. Additionally, Bernstein et al. demonstrated that infliximab improved BMD in patients suffering from CD [33]. Figure 2 presents risk factors of osteoporosis.

## 4. Micronutrients in the Diet of IBD Patients

### 4.1. Zinc

Zinc (Zn) is a bone component and participates in bone turnover and metabolism [34]. It also affects the synthesis of collagen and the activity of alkaline phosphatase [35]. Both in vitro and in vivo studies show zinc is an anabolic factor for bone. Additionally, Zn stimulates bone formation and inhibits resorption of bone, leading to an increased bone mass [36]. Primary sources of zinc are meat, nut, bean and wholegrain products; however, the absorption of Zn from plant products is lower than from animal products [37].

Zinc deficiency occurred in 35% of children and adolescents at the time of the IBD diagnosis. Nevertheless, one-year supplementation of Zn did not improve Zn levels in all patients [38]. Furthermore, among adults suffering from IBD, the level of Zn was decreased in 68% of patients (normal range is estimated at 10–17 mmol/L) [39]. As a meta-analysis demonstrated, Zn concentration was significantly lower in patients with an autoimmune disease when compared to the control group [40]. In addition, many patients have to avoid nut, bean, and wholegrain products due to the occurrence of gastrointestinal symptoms after consumption.

Xiong et al. reported that an odds ratio of phalangeal osteoporosis was higher among individuals in the first zinc intake quartile than in the second, third and fourth. Moreover, Zn intake was negatively correlated with the risk of phalangeal osteoporosis in the entire population and men, but not in women [41]. Zinc intake and the serum concentration was decreased in men with lumbar spine and femoral neck osteoporosis when compared to the groups without osteoporosis. BMD of the femoral neck, lumbar spine and the distal wrist bones was significantly lower in the group in the lowest plasma zinc quartile [42]. Additionally, the zinc level was decreased among women with osteoporosis [43]. According to Mutly et al., Zn concentration in postmenopausal women who reported osteoporosis was significantly decreased in comparison with women suffering from osteopenia or with a normal bone mass [44]. Moreover, a six-month treatment with calcitonin increased Zn levels, which allowed to formulate the conclusion that the Zn level may be used for the evaluation of the osteoporosis therapy [43].

### 4.2. Copper

Copper (Cu) participates in various enzymatic process, nucleic acid synthesis, iron metabolism and immune system functions. Moreover, as a cofactor of antioxidant enzymes, Cu removes bone free radicals, leading to an increase of the osteoblasts activity [45]. It also affects bone formation and mineralization [35] and is responsible for lysine crosslink formation in elastin and collagen by means of the activation of lysyl oxidase [45]. Additionally, Cu is a cofactor of many enzymes in collagen synthesis [46]. Since it constitutes such a vital element, its deficiency may lead to disorders in bone and cholesterol metabolism [47], as well as contributing to the development of osteoporosis [48]. On the other hand, an excessive intake of Cu may induce oxidative stress, reduce cell proliferation and damage DNA [49]. Meat, seafood, nuts and grains are the primary sources of dietary copper [46].

Głąbska et al. demonstrated a lack of significant differences in Cu intake between men with UC and healthy men [50]. Moreover, the Cu concentration was not different among UC patients and healthy subjects [51]. On the other hand, serum level of copper among children with CD was significantly decreased in comparison with the healthy children [52].

Furthermore, a lack of differences in the Cu level was observed between postmenopausal women with osteoporosis, osteopenia and normal bone mass [44]. Another study revealed that copper concentration was significantly lower among postmenopausal women with osteoporosis than in women with normal BMD [43]. The intake of Cu in patients with tooth wear did not differ from the healthy individuals, although it decreased the lumbar spine BMD in the control group. In contrast, Cu content in enamel was significantly decreased in the study group than in the control group, but no difference in the Cu serum concentration level was found between the groups [53]. A decreased Cu serum level was associated with a significantly lower BMD of the femur and the femoral neck. Moreover, a high concentration of copper was connected with an increased prevalence of fractures, particularly among men [47].

However, research studies regarding the connection between copper and BMD are not well known and further research is necessary.

### 4.3. Selenium

Selenium (Se) is a component of over 25 selenoenzymes. Se deficiency can affect increased growth and bone metabolism. Additionally, Se is an antioxidant which reduces inflammation, and affects the proliferation and differentiation of bone cells. Selenium appears in food as selenomethionine, selenocysteine and Se-methylselenocysteine [54]. Content of Se in food is various and depends on the feed supplied to the animals (in animal products), including salmon, eggs, chicken, milk products, whereas in plant products, such as brazil nuts and garlic, Se content may be influenced by the substrate in which the plants are grown [55,56].

The serum level of selenium was significantly lower among patients with UC than in the healthy subjects [51]. The concentration of Se in pediatric patients suffering from CD or UC was significantly lower when compared to the healthy children [52]. Moreover, selenium deficiency occurred in 30% of IBD patients [57].

In terms of ageing men, Se level was positively correlated with BMD [58]. As Wang et al. showed, the elderly and ageing persons with a low selenium intake (≤ 29.2 μg/day) more frequently present with osteoporosis [59]. On the other hand, there was a lack of difference in Se concentration between the postmenopausal women with osteoporosis, osteopenia and normal BMD [60]. Odabasi et al. also reported that no difference in the serum level of Se was observed between subjects with and without osteoporosis [61]. Additionally, among women over 51 years of age, if calcium consumption was lower than 800 mg/day, a high intake of Se negatively affected bone mass [62].

The data concerning the impact of Se on bone are unclear and requires further research.

### 4.4. Iron

The most common consequence of iron (Fe) deficiency is anemia, although a decreased level of Fe may result in many disorders [63]. In fact, Fe affects collagen bone matrix synthesis and is a cofactor in the enzyme responsible for the metabolism of vitamin D [35]. Therefore, iron deficiency influences bone homeostasis. In contrast, iron overload may also lead to the development of osteoporosis, for instance by an increase in the reactive oxygen spices [64]. Products which are the sources of Fe can be divided into heme (meat, fish) and non-heme (grain, legumes, vegetables, fruits) [63].

Both the deficiency and the excess of iron can lead to the weakening of bones. Low concentration of Fe causes an increase in the expression of fibroblast growth factor 23 (FGF23) gene [65]. Moreover, iron is also a metal which may catalyze a formation of reactive oxygen spices [66], as well as affecting the differentiation and the activity of osteoblasts and osteoclasts [64].

Iron deficiency anemia affects over 19% and 21% of patients with CD and UC, respectively [67]. Nevertheless, iron deficiency was also diagnosed in 37% of IBD patients without anemia [68]. In fact, no difference in the intake of Fe was observed between men suffering from UC and the healthy controls [50]. However, the intake of total iron and heme iron was not associated with risk of UC and CD development [69].

Among postmenopausal women using hormone replacement therapy, the intake of iron was linked to positive changes in BMD of Ward’s triangle and trochanter [70]. Additionally, Fe intake was associated with a greater BMD of the lumbar spine (L2-L4), trochanter, femur neck, Ward’s triangle and the total body mass in non-smoking postmenopausal women [71]. Additionally, the prevalence of low BMD decreased according to the quartiles of hemoglobin in the healthy (without anemia) individuals over 60 years of age [72]. Ferritin concentration was positively correlated with BMD of the total lumbar spine, total femur and femur neck in men, but not in women [73]. On the other hand, Kim et al. reported that both the male and female patients in the first hemoglobin quartile presented a significantly faster loss of bone mass in the femur and the femoral neck [66].

### 4.5. Cadmium

Cadmium (Cd) may inhibit bone formation and mineralization, affect the collagen matrix, as well as increase urine calcium excretion [45]. Food products (such as seafood, meat, vegetables, grains and rice), cigarette smoke and the environment can constitute sources of cadmium. In fact, cadmium affects bone health, as it disrupts the metabolism of calcium and vitamin D in the intestines and kidneys [74,75]. Interestingly, a high concentration of cadmium occurs mainly in the industrial areas [45].

The risk of osteoporosis and fractures was 32% higher in individuals with a high Cd daily intake (≥13 μg/day, median) and 31% higher in subjects with a low intake (<13 μg/day) [76]. On the other hand, there was no association between BMD and cadmium intake (median of the intake was 25.29 μg/day) in postmenopausal women [77]. Moreover, BMD of the forearm was negatively correlated with cadmium excreted with urine, which suggests a dose–effect relationship between the Cd dose and BMD [78].

An excess intake of cadmium negatively affects the bone tissue. Therefore, in order to reduce cadmium absorption, patients should avoid products (especially vegetables) from industrial regions. Furthermore, an important element in the reduction of Cd supply is avoiding smoking.

### 4.6. Silicon

Silicon (Si) participates in a cross-link between collagen and proteoglycans formation, and may also participate in the process of electrochemical bone mineralization. Additionally, Si affects bone mineral density, although the exact mechanism has not been well understood [79].

Silicon (Si) is a non-metal which may be delivered to the human body with drugs, cosmetics, medical implants, water and food. The primary nutritional sources of Si are plant products (cereals, grains, some fruit and vegetables), dairy products and meat. As studies concerning tissues and osteoblasts have shown, Si increases dry bone mass, collagen and calcium content, and elevates the proliferation of trabecular cells [80].

Silicon intake correlated positively with BMD of four hip sites in men and premenopausal women, but not in postmenopausal women. Additionally, no association was observed between the lumbar spine BMD and Si intake [81]. A 12-week long supplementation with silicon-rich (86 mg/L) water did not alter the level of type 1 cross-linked N-telopeptide, procollagen type I intact, N-terminal propeptide, bone specific alkaline phosphatase and osteocalcin [82]. Nevertheless, an animal study has shown that Si supplementation affects BMD of the femur positively, although it does not change the concentration of alkaline phosphatase and osteocalcin [83].

### 4.7. Fluorine

Fluorine (F) may interact with the bone mineral matrix. Sodium fluoride has an anabolic effect, leading to an increase in bone mass; however, the mechanism of this action remains unknown. An in vitro study has shown that the impact of fluorine on osteoclasts depends on the concentration (15–30 mg/L) and leads to a decreased osteoclasts activity, whereas a concentration of 1 mg/L increases the activity of osteoclasts. Additionally, the narrow window of the therapeutic and toxic effect of fluorine makes it difficult to investigate the mechanism of fluorine impact on the bone tissue [84]. Additionally, the fluoride anion may change the crystalline structure in the bone tissue, since fluoride stimulates the formation of bone [85]. Products rich in fluoride comprise black and green tea, seafood and wine [86].

Research suggests that adding sodium fluoride to the supplementation of calcium and vitamin D did not increase osteocalcin level and did not decrease osteoprotegerin among patients suffering from CD [87]. A study by Abitbol et al. revealed that BMD of the lumbar spine increased in osteoporotic CD patients following the supplementation of Ca and vitamin D with and without the addition of fluorides. Moreover, no significant differences in BMD between groups were found [88].

The serum level of fluoride was not associated with BMD and the incidents of osteoporotic fractures in the course of four years of observations [89]. A meta-analysis showed that depending on the duration of the therapy, the treatment with fluoride elevated BMD of the spine and hip, although it did not affect the risk of hip and spine fractures. Additionally, a dose of ≤20 mg/day of fluoride equivalents was linked with a significantly decreased risk of fractures [85]. Phipps et al. investigated the impact of the consumption of fluorinated (continuous exposure) and non-fluorinated (no exposure) water on BMD. Continuous exposure resulted in a higher BMD of the lumbar spine, femoral neck and trochanter in the exposed women, whereas a smaller radius was observed in the same group as compared with the women who were not exposed. Moreover, women who consumed fluorinated water presented a lower risk of hip fractures and vertebral fractures by 31% and 27%, respectively, in comparison to women who consumed non-fluorinated water [90].

## 5. Gut Microbiota and Micronutrients

Studies have shown that many micronutrients participate in the bacterial colonization of the intestines. One of the elements affecting gut microbiota is selenium. A deficiency and excess of Se are linked to metabolic complications and increase the risk of developing certain neoplasms [11]. The impact of Se on gut microbiota depends on the dose. In fact, a daily supplementation 0.1–2.25 μg/kg increased the variety of gut microbiota [91], whereas a dose of 0.4 mg/kg increased the number of *Akkermansia* and *Turicibacter*, and a decreased the amount of *Dorea* and *Mucispirillum* [92].

The impact of zinc on the intestinal microbiota in patients suffering from IBD is crucial. Zn is indispensable for the integrity of the intestinal epithelium. On the other hand, an excess of Zn may negatively affect microbiota by increasing the amount of *Clostridium* and *Enterococcus*. The animal study demonstrated that the supplementation of Zn increased the number of *Lactobacillus* and decreased the amount of harmful bacteria, including Salmonella sp. [93,94]. Chronic zinc deficiency causes a decline in the number and diversity of *Firmicutes*, and leads to a decrease in the SCFA production [95].

Data regarding the impact of iron on the gut microbiota content are ambiguous, and the effect probably largely depends on the dose. The supplementation of Fe for the deficiency treatment decreased the number of the beneficial microorganisms, simultaneously increasing the amount of harmful intestinal microbes. Furthermore, dysbiosis elevated intestinal inflammation [96]. On the other hand, iron supplementation in smaller doses (50 mg/day in 4 days/week for 38 weeks) did not change the significant concentration of a dominant group of bacteria (both beneficial and harmful) in the intestine among children [97]. The intake of grains enriched in Fe decreased the number of *Bifidobacteriacea* (51% vs. 37%) and increased *Bacteroidetes* (5% vs. 14%) in the stool samples [98]. A decreased number of *Bifidobacterium* in the stool sample was more significant among children with a higher dose (6.4 mg/day) of Fe supplementation than the lower dose (1.2 mg/day). An excess of Fe has been associated with an increase in *Defluviitaleaceae*, *Ruminococcaceae* and *Coprococcus* and a decrease in *Lachnospiraceae* and *Allobaculum* [99]. A higher dose of iron was also associated with an elevated virulence of the pathogenic bacteria [100]. Interestingly, a dose of less than 60 mg/day did not change the composition of the stool microbiome among women with overweight and obesity in early pregnancy [101]. Contrary to the use of supplementation, a diet with a higher content of iron resulted in an increased amount of *Bifidobacterium* among Japanese women [102]. Nevertheless, the type of supplemented iron also constitutes an important factor. A study showed that non-heme iron increased the amount of *Firmicutes*, whereas heme iron decreased the amount of *Firmicutes* [103]. Additionally, the oral intake of iron resulted in a decreased number of *Fecalibacterium prausnitzii*, *Ruminococcus bromii*, *Collinsella aerofaciens* and *Dorea*, when compared with the intravenous administration. Moreover, the administration in drops (a standard dose) may decrease the relative abundance of lactobacilli, simultaneously increasing the susceptibility to bacterial infections. Table 1 summarizes the information concerning the role of microbiota in the composition of gut microbiota.

## 6. Summary

**Figure 3:**
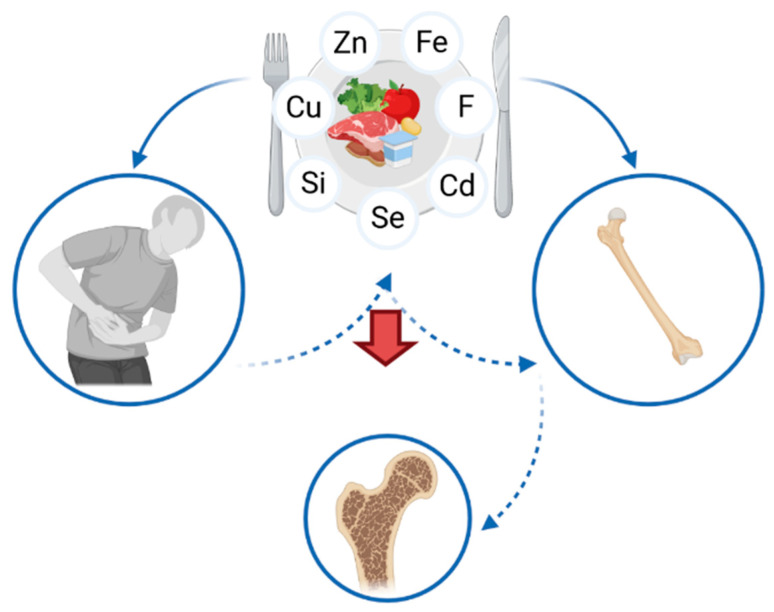
Association between the intake of micronutrients, inflammatory bowel diseases and osteoporosis.

The prevention of osteoporosis in patients suffering from IBD is an important element of medical care. A proper diet, preventing a deficiency of various nutrients, including micronutrients (Table 2), is one of the factors involved in the prevention of bone mineral density loss. Nutritional education needs to be focused on preventing both the gastrointestinal discomfort, as well as the consequences of IBD, including osteoporosis. Patients ought to be educated with regard to nutrition, taking into account the sources of calcium and vitamin D, but also of zinc, copper, selenium, iron, silicon and fluoride. Nevertheless, many patients have to eliminate certain products (such as grains or beans, which are sources of microelements) due to gastrointestinal symptoms, e.g., abdominal pain or diarrhea (Figure 3). Therefore, it is vital to emphasize the fact that an elimination diet increases the risk of microelements deficiency, leading to an elevated risk of osteoporosis, which often remains undiagnosed. Thus, patients may present nutritional deficiency, despite clinical remission and healing of the mucosa.

Therefore, patients with a chronic elimination diet should be subject to screening, including micronutrients status testing. Additionally, patients suffering from IBD ought to be educated with regard to nutrition, since a well-balanced personal diet is one of the most essential components in the prevention of both malnutrition and nutritional deficiency. Moreover, the cooperation between gastroenterologists, dieticians and other specialists is vital for comprehensive patient care and the prevention of complications, including osteoporosis.

Nevertheless, further studies concerning the role of micronutrients in the development of osteoporosis in patients suffering from IBD are necessary.

## Figures and Tables

**Table 1:** The impact of the selected micronutrients on gut microbiota.

Supplemented Micronutrients	Strain Growth	Reduction of Stains	Reference
Selenium	Akkermansia Turicibacter	Dorea Mucispirillum	Zhai, Q. [92]
Zinc	Lactobacillus	Salmonella sp.	Starke, I.C. [93] Shao, Y. [94]
Iron	Bacteroidetes * Bifidobacterium * Firmicutes	Bifidobacteriacea * Firmicutes	Qasem, W. [98] Seura, T. [102] Martin, O.C.B. [103]

* products enriched in iron (no supplementation).

**Table 2:** Summary of connection between micronutrient, their deficiency in IBD and association with osteoporosis.

Micronutrient	RDA for an Adult [104]	Food Sources	Deficiency in IBD	Association with Osteoporosis
Zinc	Women—8 mg/d Men—11 mg/d	meat, nuts, beans and whole grain products	Santucci et al., 2014 [38] Naber et al., 1998 [39] Sanna et al., 2018 [40]	Xiong, et al., 2019 [41] Hyun, et al., 2004 [42] Gür, et al., 2002 [43] Mutlu, et al., 2007 [44]
Copper	900 mg/d	meat, seafood, nuts and grain	Ojuawo et al., 2002 [52]	Beukhof, et al., 2016 [58] Wang, et al., 2019 [59]
Selenium	55 mg/d	plant and animal products (the content of Se in food is various)	Poursadegh et al., 2018 [51] Ojuawo et al., 2002 [52] Han et al., 2017 [57]	Beukhof, et al., 2016 [58] Wang, et al., 2019 [59]
Iron	Men—8 mg/d Women—18 mg/d (8 mg/d for women over 51 years old)	heme (meat, fish) and non-heme (grain, legumes, vegetables, fruits)	Madanchi et al., 2018 [67] González Alayón et al., 2018 [68]	Maurer, et al., 2005 [70] Harris, et al., 2003 [71]
Cadmium	No data	seafood, meat, vegetables, grain and rice	No data	Engström, et al., 2012 [76] Zhu, et al., 2004 [78]
Silicon	No data	plant products (cereals, grains, some fruit and vegetables), dairy products and meat	No data	Jugdaohsingh, et al., 2004 [81] Bae, et al., 2008 [83]
Fluorine *	Men—4 mg/d Women—3 mg/d	black and green tea, sea food and wine	No data	Vestergaard, et al., 2008 [85] Phipps, et al., 2000 [90]

* Adequate Intake.

## Data Availability

Statement excluded.
